# Human MLL-AF9 Overexpression Induces Aberrant Hematopoietic Expansion in Zebrafish

**DOI:** 10.1155/2018/6705842

**Published:** 2018-05-30

**Authors:** Jiaqi Tan, Lei Zhao, Gaoxiang Wang, Tongjuan Li, Dan Li, Qian Xu, Xing Chen, Zhen Shang, Jue Wang, Jianfeng Zhou

**Affiliations:** ^1^Department of Hematology, Tongji Hospital, Tongji Medical College, Huazhong University of Science and Technology, Wuhan 430030, China; ^2^Cancer Biology Research Center, Tongji Hospital, Tongji Medical College, Huazhong University of Science and Technology, Wuhan 430030, China

## Abstract

The 11q23 of the mixed lineage leukemia 1 (*MLL1*) gene plays a crucial role in early embryonic development and hematopoiesis. The* MLL-AF9* fusion gene, resulting from chromosomal translocation, often leads to acute myeloid leukemia with poor prognosis. Here, we generated a zebrafish model expressing the human* MLL-AF9* fusion gene. Microinjection of human* MLL-AF9* mRNA into zebrafish embryos resulted in enhanced hematopoiesis and the activation of downstream genes such as* meis1 *and* hox *cluster genes. Embryonic MLL-AF9 expression upregulated HSPC and myeloid lineage markers. Doxorubicin and MI-2 (a menin inhibitor) treatments significantly restored normal hematopoiesis in MLL-AF9-expressing animals. This study provides insight into the role of MLL-AF9 in zebrafish hematopoiesis and establishes a robust and efficient in vivo model for high-throughput drug screening.

## 1. Introduction

Chromosomal translocations of 11q23 of the mixed lineage leukemia gene (known as* KMT2A*,* MLL*, or* HRX*) account for 5–10% of all acute leukemia cases, including acute lymphoid, myeloid, and biphenotypic leukemia, and are generally associated with inferior prognosis [1]. Particularly, the t(19;11)(p22;q23) reciprocal translocation, which generates the* MLL-AF9* fusion gene, often leads to myelomonoblastic acute myeloid leukemia (AML) with a high risk for poor outcome [2]. Although several treatment approaches including targeted therapies have been developed, MLL translocation leukemia remains a difficult clinical challenge [3].

The wild-type MLL protein contains a conserved SET domain and possesses histone H3 lysine 4 (H3K4) methyltransferase activity. The wild-type MLL protein can be processed into two fragments (MLL-C and MLL-N) by taspase 1, which associate with different partners (menin, LEDGF, WDR5, ASH1/2, HCF2, RBBP5, MOF, etc.) and assemble into sophisticated complexes [4]. Under normal conditions, MLL plays a crucial role in regulating the transcriptional activation of* HOX *cluster genes to promote early embryonic development and organogenesis, including hematopoiesis [5, 6]. When fused with other genes, the SET domain is cleaved off from the N-terminus, and the residual part of MLL-N is directly linked with the fusion partner [7]. In comparison with wild-type proteins, MLL fusion proteins are associated with deregulated target gene expression. Specifically, oncogenic MLL proteins promote and maintain the overexpression of* MEIS1 *and* HOX *cluster genes, triggering the pathological development of MLL-associated leukemia [8, 9].

To address the underlying mechanisms and explore possible therapeutic interventions for MLL-AF9-associated leukemia, various murine models have been generated to recapitulate the human disease, which exhibit the phenotypes of myeloproliferative disorders or myeloid leukemia [10–12]. Although murine models have provided key insights into disease pathogenesis, there is still a lack of a feasible and robust vertebrate platform for efficient large-scale chemical screening at the whole-organism level to identify agents with therapeutic potential.

Zebrafish has emerged as a reliable and convenient model to study hematopoiesis and human leukemia. The genetic programs of hematopoiesis are highly conserved between zebrafish and humans, and the abundant and transparent embryos of zebrafish allow studies of hematopoiesis at early timepoints [13, 14]. Previous studies have reported that zebrafish orthologs exhibit 68–100% sequence similarity to human* MLL* gene in functional domains, and zebrafish mll promotes the expression of* hox *genes that regulate zebrafish primitive and definitive hematopoiesis [15, 16]. However, how oncogenic* MLL* fusion genes affect early hematopoietic development in zebrafish remains unclear. In this study, we attempted to elucidate the role of MLL-AF9 in zebrafish hematopoiesis. The zebrafish model expressing MLL-AF9 recapitulated the myeloproliferative disorders observed in murine models and human disease. Therefore, the zebrafish model may be a suitable in vivo platform for chemical screening.

## 2. Materials and Methods

### 2.1. Ethics Statement

All experiments described in this study were performed in accordance with the guidelines of the Institutional Committee of Animal Care and Treatment of Tongji Hospital and were approved by the Ethics Committee of Tongji Hospital.

### 2.2. Maintenance of Zebrafish

Wild-type zebrafish (*Danio rerio*) (strain AB) was maintained under standard conditions, and embryos were staged according to standard procedures [17, 18].

### 2.3. Plasmid Construction

The pMSCV-neo-MLL-AF9 plasmid was a gift from Dr. Michael Cleary (Stanford University). A primer pair was designed to amplify only the MLL-AF9 cDNA sequence without the promoter from the pMSCV-neo-MLL-AF9 plasmid by polymerase chain reaction (PCR) using Phanta Super-Fidelity DNA Polymerase (Vazyme Biotechnology, Nanjing, China). The primers used are listed in Supplementary Table 1. To generate a vector for in vitro transcription with the T3 promoter, the PCR product was purified (QIA quick PCR Purification Kit; QIAGEN) and cloned into an empty vector (pCMV-C-Flag Plasmid; Beyotime Biotechnology) with the T3 promoter and a polyA signal sequence (In-Fusion HD Cloning Kits). The generated vector was designated T3-MLL-AF9-polyA.

### 2.4. Synthesis of mRNA

The T3-MLL-AF9-polyA plasmid was linearized by PCR using Phanta Super-Fidelity DNA Polymerase (Vazyme Biotechnology) and purified (QIA quick PCR Purification Kit; QIAGEN). Then, mRNA was synthesized with T3 RNA polymerase (mMESSAGEmMACHINE T3 Kit; Ambion).

### 2.5. Microinjection and Overexpression of Human MLL-AF9 in Zebrafish

MLL-AF9 mRNA was diluted to working concentrations with sterilized H_2_O. To determine the optimal concentration, a concentration gradient (100, 150, 200, and 250 ng/*μ*L) was used for microinjection, and MLL-AF9 mRNA was injected into 1–2-cell stage embryos in the yolk sac with an injection volume of approximately 5nL. The morphology of the injected embryos was observed at 24, 48, and 72h after fertilization (hpf).

### 2.6. Quantitative Reverse Transcription PCR (qRT-PCR)

Total RNA was extracted from zebrafish larvae using TRIzol reagent (T9424; Sigma-Aldrich). RNA quantification was performed using a NanoDrop microvolume spectrophotometer (Thermo Fisher). RNA was reverse transcribed into cDNA using PrimeScript™ RT Reagent Kit (PR037A; TaKaRa Bio Inc., Kusatsu, Japan). qPCR was performed using a CFX96 Touch™ Real-Time PCR Detection System (Bio-Rad). The PCR primers were either described in previous studies [19] or listed in Supplementary Table 1. The target genes were normalized to *β*-actin.

### 2.7. In Situ Hybridization

Digoxigenin-labeled (Roche) antisense RNA probes for specific hematopoietic transcription factors (lmo2, gata1, mpo, lyz, l-plastin, fli, and c-myb) were transcribed from cDNA. PCR primers were either used in previous studies [16, 19] or listed in Supplementary Table 1. Whole-mount in situ hybridization (WISH) was performed as previously described [20].

### 2.8. Isolation of Hematopoietic Cells from Zebrafish Embryos and Cytospin Preparation

At 48 hpf, 30 embryos were anesthetized with 0.016% tricaine and placed in PBS. Embryonic tails were removed, and the cells were collected by centrifugation at 800 rpm for 5 min and subjected to Wright's staining.

### 2.9. Western Blotting

Zebrafish embryos were collected at 36hpf and ground to obtain single cells. The cells were lysed in SDS buffer with a cocktail of protease inhibitors. Total protein was extracted and denatured. Then, 50 *μ*g of each sample was loaded on a SDS-PAGE gel, and the presence of the MLL protein was detected using Abcam antibody (ab52099 Abcam, Cambridge, UK), which could recognize the N-terminal sequences of the human wild-type MLL protein. GAPDH antibody (Sigma-Aldrich) was used as a control.

### 2.10. Drug Administration

Drugs were dissolved in DMSO and diluted to different concentrations. DMSO or the indicated doses of drugs (all compounds were obtained from Sigma-Aldrich, St. Louis, MO, USA) were dissolved in egg water (to avoid toxicity, the final DMSO concentration was maintained at<0.1%). Every 25–30 embryos were randomly grouped and arrayed in 6-well plates with 6 mL of drug-added egg water. The embryos were then incubated at 28.5°C.

### 2.11. Statistical Analysis

Data are presented as the mean ± standard error of the mean (SEM) and were analyzed with GraphPad Prism 6 for Windows (GraphPad Software) using Student's t-test. ^*∗*^P < 0.05; ^*∗∗*^P < 0.01; ^*∗∗∗*^P < 0.001.

## 3. Results

### 3.1. Overexpression of Human MLL-AF9 in Zebrafish

To generate the zebrafish model expressing MLL-AF9, MLL-AF9 mRNA was injected into zebrafish embryos at various concentrations (100ng/*μ*L, 150ng/*μ*L, 200ng/*μ*L, and 250ng/*μ*L). In comparison with uninjected wild-type embryos, embryos injected with MLL-AF9 mRNA at concentrations of 100ng/*μ*L, 150ng/*μ*L, and 200ng/*μ*L showed a similar morphology. In embryos injected with 250ng/*μ*L MLL-AF9 mRNA, we observed a short body axis and increased hematopoietic cells (data not shown).

**Figure 1:**
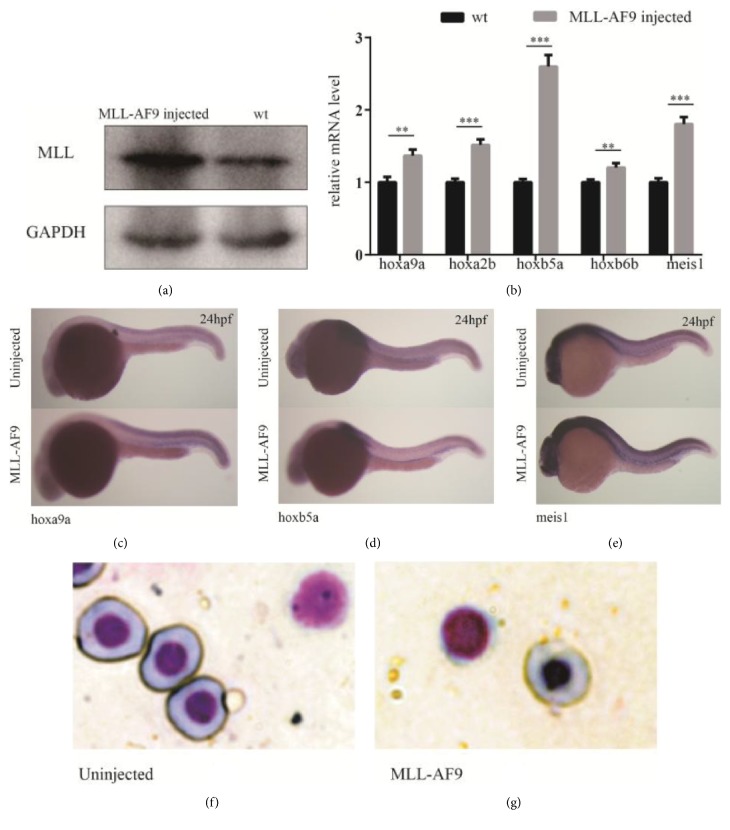
**MLL-AF9 overexpression in zebrafish embryos and activation of MLL downstream genes**. MLL-AF9 mRNA was injected into zebrafish at the 1–2-cell stage (around 5ng per embryo). Injected embryos and control embryos were harvested at 48hpf. (a) Western blotting of the N-terminus of the MLL protein in MLL-AF9-injected and control embryos. (b) Relative expression of MLL downstream genes analyzed by qRT-PCR. (c–e) WISH assays of hoxa9a, hoxb5a, and meis1 in MLL-AF9-injected and control embryos at 24hpf. (f, g) Morphology of the hematopoietic cells of control embryos and MLL-AF9-injected embryos at 48 hpf.

At 24 and 48hpf, MLL-AF9-injected embryos were harvested, and their RNA was extracted. Embryos injected with 100ng/*μ*L and 150ng/*μ*L MLL-AF9 mRNA showed no obvious variation in hematopoietic markers (data not shown), whereas embryos injected with 200ng/*μ*L MLL-AF9 mRNA showed an increase in hematopoietic markers. Western blotting revealed a significant increase in the MLL protein in embryos injected with 200ng/*μ*L MLL-AF9 mRNA (Figure 1(a)), demonstrating the successful translation of human MLL-AF9 mRNA in zebrafish. Therefore, a mRNA concentration of 200ng/*μ*L was used for subsequent injections.

To evaluate the effect of MLL-AF9 on the morphology of zebrafish hematopoietic cells, we isolated hematopoietic cells from zebrafish embryos at 48hpf and observed them under a microscope following Wright's staining. There were no noticeable changes in the morphology of hematopoietic cells (Figures 1(f) and 1(g)).

### 3.2. Human MLL-AF9 Upregulation of Hox Cluster and Meis1 Gene Expression in Zebrafish

A previous study has demonstrated the essential role of MLL in maintaining the expression of selected* HOX *cluster genes during embryonic development [7]. Furthermore,* HOXA9 *and* MEIS1 *have been reported as target genes of oncogenic MLL-AF9 fusion proteins in human leukemia [6, 21]. Therefore, we determined whether human MLL-AF9 could affect the expression of similar genes in zebrafish. qRT-PCR was performed to evaluate the expression level of* hoxa9a*,* hoxa2b*,* hoxb5a*,* hoxb6b*, and* meis1 *genes. Consistent with the results obtained from human leukemia cells and mouse models, a significant upregulation of* hox *cluster and* meis1 *gene expression at 48 hpf in MLL-AF9-expressing embryos was observed (Figure 1(b)). Furthermore, we performed in situ hybridization to confirm whether MLL-AF9 could induce the expression of* meis1* and* hox* cluster genes such as* hoxa9a* and* hoxb5a* in hematopoietic regions (Figures 1(c)–1(e)). These results suggest a highly conserved transcriptional program shared by mammals and zebrafish with regard to MLL complexes.

### 3.3. Effect of Human MLL-AF9 on Zebrafish Primitive and Definitive Hematopoiesis

Two waves of hematopoiesis, i.e., primitive and definitive hematopoiesis, occur during zebrafish development [22, 23]. To determine the effect of MLL-AF9 on zebrafish hematopoiesis, we examined both primitive and definitive hematopoietic cell markers.

**Figure 2:**
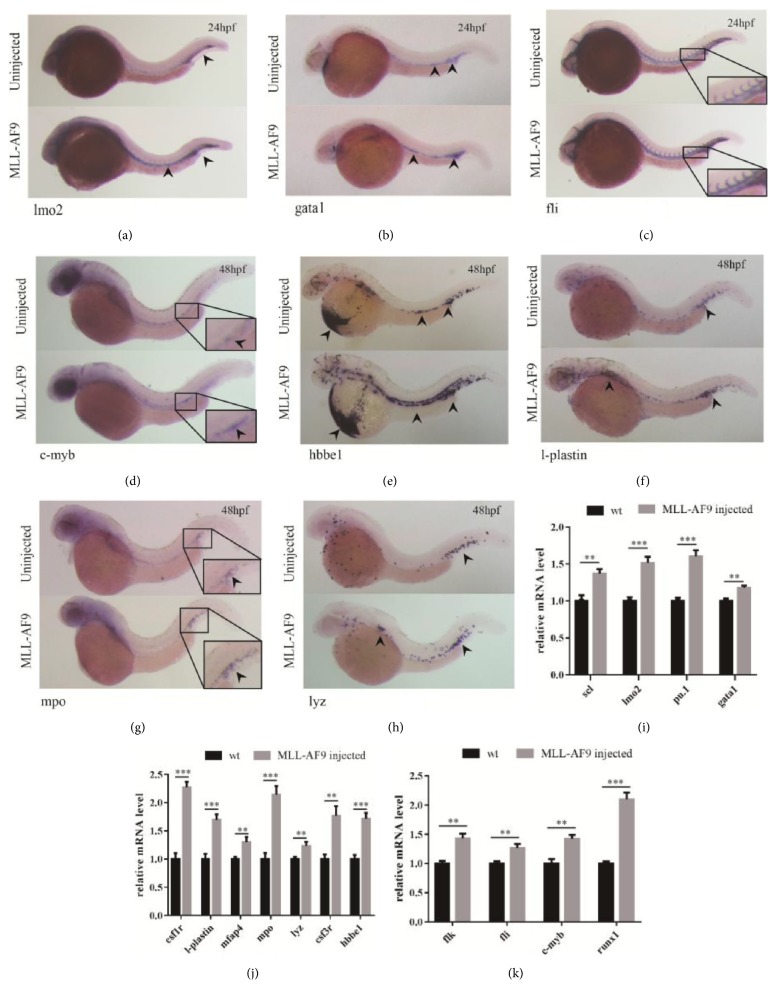
**Induction of zebrafish primitive and definitive hematopoiesis by MLL-AF9**. WISH assays and qRT-PCR analysis of hematopoietic markers in MLL-AF9-injected and control embryos. (a–c) WISH assays of hematopoietic markers (lmo2 and gata1) and a vasculature formation marker (fli) in MLL-AF9-injected and control embryos at 24hpf. (d–h) WISH assays at 48hpf of the definitive hematopoietic stem cell marker c-myb, the erythropoietic marker hbbe1, and the myeloid lineage markers l-plastin, mpy, and lyz. Relative expression of the marker genes for (i) primitive hematopoiesis at 24hpf, (j) hematopoiesis at 48hpf, (k) vasculature formation (flk, fli) at 24hpf, and (k) definitive hematopoiesis initiation (c-myb and runx1) at 48hpf analyzed by qRT-PCR.

The expression of scl and lmo2 indicates the formation of primitive progenitors mainly in two regions, the intermediate cell mass (ICM) and rostral blood island (RBI), reflecting primitive hematopoiesis in zebrafish [14, 23]. At 24hpf, scl, and lmo2 were slightly upregulated in MLL-AF9-injected embryos (Figures 2(a) and 2(i)), indicating an enhancement in zebrafish primitive hematopoiesis. Moreover, we detected a slight upregulation of gata1 (a critical transcription factor of primitive erythropoiesis expressed in the ICM) and a significant overexpression of the myeloid progenitor marker pu.1 [24] in MLL-AF9-injected embryos at 24hpf (Figures 2(b) and 2(i)), indicating that MLL-AF9 could induce both primitive erythropoiesis and myeloid hematopoiesis.

Zebrafish definitive hematopoietic stem cells (HSCs), capable of unlimited self-renewal and generating all hematopoietic lineages, arise de novo from the ventral wall in the aorta-gonad-mesonephros region, as demonstrated by the expression of c-myb and runx-1 at around 26–28 hpf. To investigate the effects of MLL-AF9 on embryonic definitive hematopoiesis, we measured the expression of runx1 and c-myb in the embryos at 48hpf. Both runx1 and c-myb were upregulated in MLL-AF9-injected embryos (Figures 2(d) and 2(k)). HSCs are derived from flk1^+^ hemogenic endothelium [25, 26]. Therefore, to determine whether MLL-AF9 could promote vasculogenesis, the endothelial markers fli and flk1 were assessed at 24hpf; a modest increase was detected (Figures 2(c) and 2(k)). These results suggest that MLL-AF9 may promote vasculature formation, which is important for HSC genesis.

We further focused on zebrafish myeloid development, as the MLL-AF9 fusion gene typically results in myelomonoblastic AML. Cell markers of monocyte/macrophage lineages (csf1r, mfap4, and l-plastin) and neutrophil granulocyte lineages (lyz, csf3r, and mpo) were analyzed. As expected, the myeloid hematopoietic cell markers were significantly upregulated at 48hpf in MLL-AF9-injected embryos (Figures 2(f)–2(j)). Taken together, these results suggest that both primitive hematopoiesis and definitive hematopoiesis were affected in the zebrafish model expressing MLL-AF9, and myeloid expansion closely recapitulated the myeloproliferative phenotypes observed in other models [10].

Although MLL-AF9 mRNA injection caused hematopoietic changes in zebrafish embryos, these effects may not be restricted to hematopoietic tissues. Therefore, to examine whether other tissues were affected by MLL-AF9 overexpression, we performed qRT-PCR and WISH to evaluate the expression of other tissue markers including the neural marker glutamate decarboxylase 1b (gad1b), the cardiac chamber marker cmlc2, the skeletal muscle marker mck, and the hepatocyte marker fabp10a at 24, 48, 48, and 96hpf, respectively. MLL-AF9 had minimal effects on cmlc2, mck, and fabp10a and slightly downregulated gad1b (Figures S1a–S1e).

### 3.4. Doxorubicin Inhibition of MLL-AF9-Induced Hematopoietic Expansion in Zebrafish

**Figure 3:**
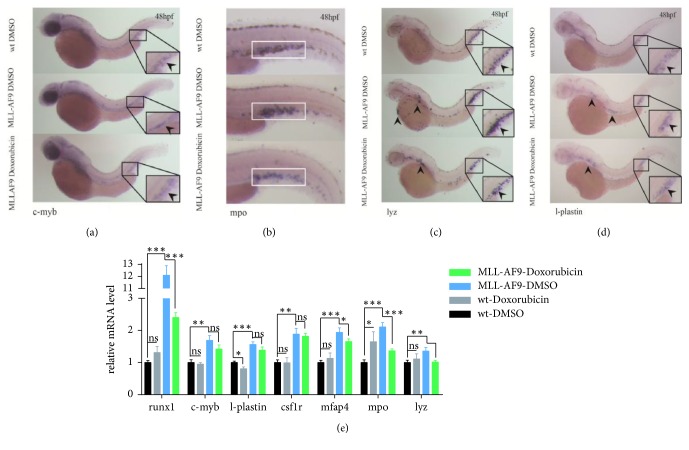
**Doxorubicin inhibition of MLL-AF9-induced hematopoiesis.** Doxorubicin and DMSO (control) were administered to MLL-AF9-injected and wild-type embryos from 24 to 48hpf. WISH assays of the (a) definitive hematopoietic stem cell marker c-myb and (b–d) myeloid lineage markers mpo, lyz, and l-plastin in wild-type embryos treated with DMSO (top), MLL-AF9-injected embryos treated with DMSO (middle), and MLL-AF9-injected embryos treated with doxorubicin (bottom) at 48hpf. (e) Relative expression of the marker genes for definitive hematopoiesis (runx1 and c-myb) and myeloid hematopoiesis (l-plastin, csf1r, mfap4, mpo, and lyz) in wild-type and MLL-AF9-injected embryos treated with DMSO or doxorubicin analyzed by qRT-PCR. Wild-type embryos treated with DMSO were used as a control.

Considering the association of MLL-AF9 with stem cell development and myeloid expansion, we attempted to determine whether a chemotherapy agent could inhibit MLL-AF9-induced hematopoietic expansion. Doxorubicin, a commonly used chemotherapy agent, acts on DNA by intercalation and inhibition of macromolecular biosynthesis [27]. AML MONOMAC-6 cells containing human MLL-AF9 have been reported to be sensitive to doxorubicin. Mice transplanted with bone marrow progenitor cells expressing MLL-AF9 have demonstrated sensitivity to doxorubicin [28]. Therefore, we administered doxorubicin to MLL-AF9-injected embryos from 24 to 48hpf to examine hematopoiesis in response to chemotherapy treatment. As shown in Figure 3, doxorubicin did not have a noticeable effect on hematopoiesis in uninjected embryos at 3*μ*mol/L. However, it significantly reduced HSC differentiation and myeloid expansion in MLL-AF9-injected embryos, as shown by the significant decrease in the stem cell marker runx1 and myeloid markers including csf1r, mfap4, mpo, and lyz at 48hpf (Figures 3(a)–3(e)). These findings provided evidence that MLL-AF9-induced hematopoietic changes were responsive to the clinical chemotherapy drug doxorubicin in zebrafish.

### 3.5. Effect of MI-2 on MLL-AF9-Induced Hematopoiesis in Zebrafish

**Figure 4:**
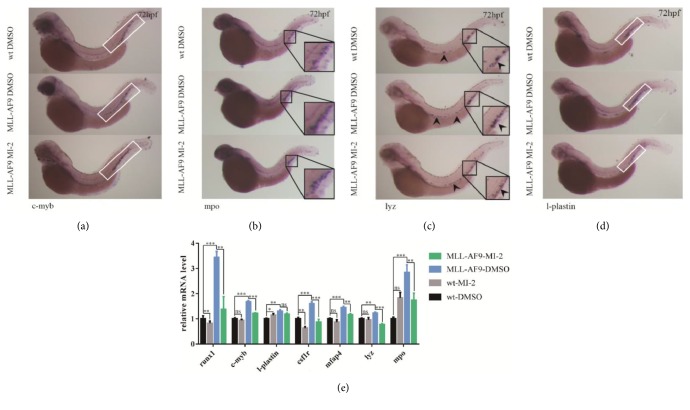
**Amelioration of MLL-AF9-induced hematopoiesis by MI-2**. The menin inhibitor MI-2 (2.5mmol/L) and DMSO (control) were administered to MLL-AF9-injected and wild-type embryos from 24 to 72hpf. WISH assays of the (a) definitive hematopoietic stem cell marker c-myb and (b–d) myeloid lineage markers mpo, lyz, and l-plastin in wild-type embryos treated with DMSO (top), MLL-AF9-injected embryos treated with DMSO (middle), and MLL-AF9-injected embryos treated with MI-2 (bottom) at 72hpf. (e) Relative expression of the marker genes for definitive hematopoiesis (runx1 and c-myb) and myeloid hematopoiesis (l-plastin, csf1r, mfap4, mpo, and lyz) in wild-type and MLL-AF9-injected embryos treated with MI-2 or doxorubicin analyzed by qRT-PCR. Wild-type embryos treated with DMSO were used as a control.

The menin inhibitor MI-2 has been reported to selectively target MLL-related genes such as* HOXA9* and* MEIS1*, and it is also effective in blocking hematopoietic expansion induced by MLL fusion genes. In human and mouse MLL-rearranged leukemia cells, the administration of menin inhibitors can lead to growth inhibition, apoptosis, and differentiation [29]. Therefore, to determine whether the menin inhibitor MI-2 could reverse the MLL-AF9-induced phenotype and restore normal hematopoiesis in zebrafish, we incubated MLL-AF9-injected embryos with MI-2 from 24 to 72hpf. Interestingly, 2.5 *μ*mol/L of MI-2 slightly inhibited the expression of runx1 and csf1r but did not have a significant effect on mfap4, mpo, and lyz at 72hpf in wild-type embryos (Figure 4(e)). On the other hand, MI-2 (at the same concentration) significantly reduced the expression of the stem cell marker runx1 and myeloid markers csf1r, mfap4, mpo, and lyz at 72hpf in MLL-AF9-injected embryos (Figures 4(a)–4(e)). Consistent with the results of previous studies carried out using human and mouse leukemia cells, which indicated that MI-2 could delay stem cell development and myeloid expansion [29], our results confirmed that hematopoietic expansion induced by MLL-AF9 was susceptible to the menin inhibitor in the zebrafish model.

## 4. Discussion

Robust and reliable animal models are essential for understanding the pathogenesis of and designing targeted therapies for MLL translocation leukemia. In this study, we generated a novel zebrafish model by microinjection of human MLL-AF9 mRNA into zebrafish embryos. We hypothesized that if the zebrafish model can recapitulate the phenotypic characteristics of MLL-AF9-driven leukemia in humans and mice, it would be responsive to chemotherapy agents and targeted drugs used in human patients. As a result, this model could serve as a highly efficient vertebrate platform for molecular pathway analysis and high-throughput chemical screening.

We have developed this model to mimic the human disease condition. Although the important functional domains of the zebrafish* mll *and human* MLL *gene are highly conserved [15, 16], whether a* MLL* fusion gene of human origin could regulate hematopoiesis in zebrafish has remained unclear. In zebrafish,* mll* knockdown could impair primitive and definitive hematopoiesis [16], similar to the hematopoietic defects observed in a heterozygous knock-out mouse model [6]. Our study is the first to demonstrate that the expression of a* MLL* fusion gene can lead to hematopoietic expansion in zebrafish, thus providing the basis for generating other oncogenic MLL fusion models in this species. In addition, human MLL-AF9 upregulated the leukemia-related oncogenes* hox *and* meis1 *in our zebrafish model, which is in agreement with observations in human cell lines and mouse models [6]. These results strongly indicated that the key signaling machinery associated with the abnormal MLL fusion protein was conserved in the zebrafish model. This model could facilitate molecular pathway analysis to understand the pathogenesis of MLL translocation leukemia.

We chose to establish the zebrafish model expressing MLL-AF9 as several murine models expressing MLL-AF9 were generated previously [10, 30–34]. In addition, MLL-AF9-induced phenotypic changes have been well validated in mice. For example, in a knock-in mouse model in which the MLL-AF9 oncogene was widely expressed in all cells throughout development, mice exhibited developmental defects and early phase myeloproliferation, which ultimately led to AML [10]. Similarly, MLL-AF9 was widely expressed in our zebrafish model. Both the knock-in mouse model and the zebrafish model expressing MLL-AF9 could cause developmental defects and myeloproliferation. These results suggest that the zebrafish model closely mimicked the phenotypic changes observed in mammals. Furthermore, with easy access to embryos, the use of this model can overcome the time-consuming and costly maintenance procedures of existing murine models.

To demonstrate the feasibility of this model in drug screening, we used the common chemotherapy drug doxorubicin and the menin inhibitor MI-2. The N-terminus of the MLL protein can bind to menin and LEDGF. Direct MLL fusion proteins such as MLL-AF9 can retain the N-terminus and thus can bind to menin. The interaction of MLL with menin is essential for MLL fusion-induced leukemia [35–37]. The menin inhibitor MI-2 has been reported to be effective in blocking hematopoietic expansion induced by MLL fusion genes in human and mouse leukemia cells. Our results demonstrated that the pharmacologic effects of both drugs were retained in MLL-AF9-expressing zebrafish embryos, thus highlighting the possibility of using the zebrafish model for drug screening.

Notably, the current zebrafish model expressing MLL-AF9 is a short-term model in which hematopoiesis can be rapidly observed. The injected MLL-AF9 mRNA can induce hematopoietic expansion in several days; however, this will not be long enough for the development of leukemia-like diseases. Similar to some of the previously established zebrafish models [38–40], oncogene mRNA injection in zebrafish would not directly lead to leukemia. Nevertheless, these models share many features with human diseases and thus may provide mechanistic insights into oncogenesis. Although the mRNA-injected model is easy to generate, a transgenic zebrafish model should be established in the future for long-term observation of MLL translocation leukemia. Additional drugs could be evaluated in this highly efficient zebrafish model to explore effective therapeutic strategies for MLL-AF9 leukemia.

## 5. Conclusion

In this study, we established a zebrafish model expressing the human MLL-AF9 oncogenic fusion protein. MLL-AF9 mRNA injection activated the expression of leukemia-related oncogenes such as the* hox *cluster and* meis1 *genes in the hematopoietic regions and aberrantly enhanced embryonic hematopoiesis including myeloid hematopoiesis in zebrafish. The administration of doxorubicin and the menin inhibitor MI-2 ameliorated MLL-AF9-induced hematopoietic expansion in MLL-AF9-expressing embryos. This model can be used as a highly efficient vertebrate platform for molecular pathway analysis and high-throughput chemical screening.

## Supplementary Materials

Supplementary Materials Original primers used in this study are listed in Supplementary Table 1. And effects of MLL-AF9 on nonhematopoietic tissues are shown in Figure S1.
