# Corrigendum to “Human MLL-AF9 Overexpression Induces Aberrant Hematopoietic Expansion in Zebrafish”

**DOI:** 10.1155/2022/9839650

**Published:** 2022-01-22

**Authors:** Jiaqi Tan, Lei Zhao, Gaoxiang Wang, Tongjuan Li, Dan Li, Qian Xu, Xing Chen, Zhen Shang, Jue Wang, Jianfeng Zhou

**Affiliations:** ^1^Department of Hematology, Tongji Hospital, Tongji Medical College, Huazhong University of Science and Technology, Wuhan 430030, China; ^2^Cancer Biology Research Center, Tongji Hospital, Tongji Medical College, Huazhong University of Science and Technology, Wuhan 430030, China

In the article titled “Human MLL-AF9 Overexpression Induces Aberrant Hematopoietic Expansion in Zebrafish” [1], there were errors in the reporting of the expression experiments.

In Figure 1 and Section 3.1 in Results, an anti-human MLL1/KMT2A antibody [2] was reported as being used to demonstrate the expression of an *in vitro* synthesized MLL-AF9 fusion transcript. The authors say that the antibody reported in the article is incorrect and the N-terminus antibody applied in this study is MLL1 (D2M7U) Rabbit mAb (Amino-terminal Antigen) from CST [3].

Previous studies show that MLL-AF9 has a molecular weight (MW) of about 170 kDa [4]. Figure 1(a) appears to show a band of a similar size in the control. However, no MLL-AF9 was in the control and only endogenous zebrafish MLL should be present, with a MW of ~490 kDa. Zebrafish MLL/KMT2A (4218 amino acids, MW 456.73 KDa) is cleaved by Taspase1 after amino acid 2917 (at QVD·GADD) resulting in a 316 kDa fragment, which may be recognized by an MLL-N terminus antibody. If there is cross-reactivity of the antibody with zebrafish MLL, then MLL-AF9, zebrafish MLL, and Taspase1-cleaved zebrafish MLL should run at different positions on the gel. The bands detected appear to be full-length zebrafish MLL (456 KDa) and Taspase1-cleaved zebrafish MLL (316 KDa), but not a band from the transgene (at ~170KDa); the band for the transgene should be somewhere below the uppermost marker band.

The authors provided the original unadjusted Western blot for MLL in Figure 1(a) and another independent replicate of the same experiment, as well as the blot for GAPDH from Figure 1(a) and the raw mRNA expression levels for Figures 1(b), 2(i)–2(k), 3(e), and 4(e) ((available here) Supplementary Materials). The marker was a previous specification of Thermo Fisher PageRuler™ 26616. In both images, the MLL-AF9 bands (indicated with a black arrow) were shown above the uppermost marker (170 kDa), along with a lighter band or spot (indicated with a red arrow) around the same size in the controls. Possible bands for zebrafish endogenous MLL proteins (indicated with a blue arrow) were also present in both groups in the replicate. The nature of the band or spot with MW ~220 kDa in the control is unclear; the light band or spot recognized by the MLL-N terminus antibody in the control may be a nonspecific cross-reaction, which happens to be at a similar size to the target band.

To confirm overexpression of human MLL-AF9 in zebrafish, the authors conducted further q-RT-PCR experiments. Detailed methods of zebrafish maintenance, grouping, and overexpression of human MLL-AF9 mRNA are described in Materials and Methods in the manuscript. A concentration of 200 ng/*μ*l of human MLL-AF9 mRNA was used for microinjection. Total RNA was extracted from zebrafish larvae at 48 hpf, 30 larvae for each test; reverse transcribed into cDNA; and used for real-time quantitative PCR. The target gene was the MLL region of human MLL-AF9, and the relative expression of the target gene was normalized to zebrafish *β*-actin. Primers used for quantitative PCR are qMA9 F1: 5′-ccactccagcttccaggaag-3′, and qMA9 R1: 5′-cagggatacttgggcggg-3′.

As shown in Figure 1 below, the mRNA-injected group has a higher expression of the human MLL-AF9 compared to the wild-type group (fold change of 758.9, *P* < 0.0001, *t*-test). As the q-RT-PCR primers are specifically designed for the MLL region of human MLL-AF9 and theoretically unable to bind the cDNA of wild-type zebrafish, this indicates overexpression of the human MLL-AF9 in the zebrafish model. The original data are presented in Table 1.

Additionally, there was an error in Figure 2(d), where the second image of Figure 3(a) MLL-AF9 DMSO was inadvertently duplicated as the second image of Figure 2(d) MLL-AF9. The corrected figure is shown below.

## Figures and Tables

**Figure 1:**
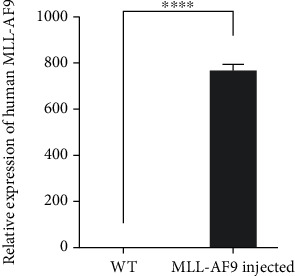
Relative expression of human MLL-AF9 in zebrafish.

**Figure 2:**
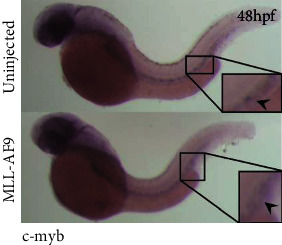
Induction of zebrafish primitive and definitive hematopoiesis by MLL-AF9. WISH assays and qRT-PCR analysis of hematopoietic markers in MLL-AF9-injected and control embryos. (a–c) WISH assays of hematopoietic markers (lmo2 and gata1) and a vasculature formation marker (fli) in MLL-AF9-injected and control embryos at 24 hpf. (d–h) WISH assays at 48 hpf of the definitive hematopoietic stem cell marker c-myb, the erythropoietic marker hbbe1, and the myeloid lineage markers l-plastin, mpy, and lyz. Relative expression of the marker genes for (i) primitive hematopoiesis at 24 hpf, (j) hematopoiesis at 48 hpf, (k) vasculature formation (flk, fli) at 24 hpf, and (k) definitive hematopoiesis initiation (c-myb and runx1) at 48 hpf analyzed by qRT-PCR.

**Table 1:** Original data of the q-RT-PCR experiment.

Gene	Sample	Cq value	Average Cq value
*β*-Actin	WT	21.16	21.27
*β*-Actin	WT	21.28	
*β*-Actin	WT	21.36	
*β*-Actin	MLL-AF9 injected	21.45	21.50
*β*-Actin	MLL-AF9 injected	21.52	
*β*-Actin	MLL-AF9 injected	21.53	
Human MLL-AF9	WT	32.31	32.34
Human MLL-AF9	WT	32.67	
Human MLL-AF9	WT	32.04	
Human MLL-AF9	MLL-AF9 injected	22.89	23.01
Human MLL-AF9	MLL-AF9 injected	23.03	
Human MLL-AF9	MLL-AF9 injected	23.1	

## Supplementary Materials

Supplementary Materials Original Western blot for MLL in Figure 1(a) in the article and an independent replicate of the same experiment. Original Western blot for GADPH from Figure 1(a). Images of the MLL1 (D2M7U) Rabbit mAb (Amino-terminal Antigen) antibody. Raw mRNA expression levels for Figures 1(b), 2(i)–2(k), 3(e), and 4(e).
