# Supplementary material for: Human MLL-AF9 Overexpression Induces Aberrant Hematopoietic Expansion in Zebrafish
Source: Biomed Res Int. 2018 May 30;2018:6705842. doi: 10.1155/2018/6705842 (PMC5998191; doi:10.1155/2018/6705842)
Supplement: Supplementary Materials — Original primers used in this study are listed in Supplementary Table 1. And effects of MLL-AF9 on nonhematopoietic tissues are shown in Figure S1. [file 6705842.f1.pdf]

Supplementary Material

Supplementary Figure 1. Effect of MLL-AF9 on non-hematopoietic tissue markers.

WISH assays of non-hematopoietic markers, including (a) the neural marker gad1b at 24hpf, (b) the cardiac chamber marker cmlc2 at 48hpf, (c) the skeletal muscle marker mck at 48hpf, and (d) the hepatocyte marker fabp10a at 96hpf in MLL-AF9-injected and control embryos. (e) qRT-PCR analysis of gad1b, cmlc2, mck, and fabp10a at 24, 48, 48, and 96hpf, respectively.

Supplementary Figure 1

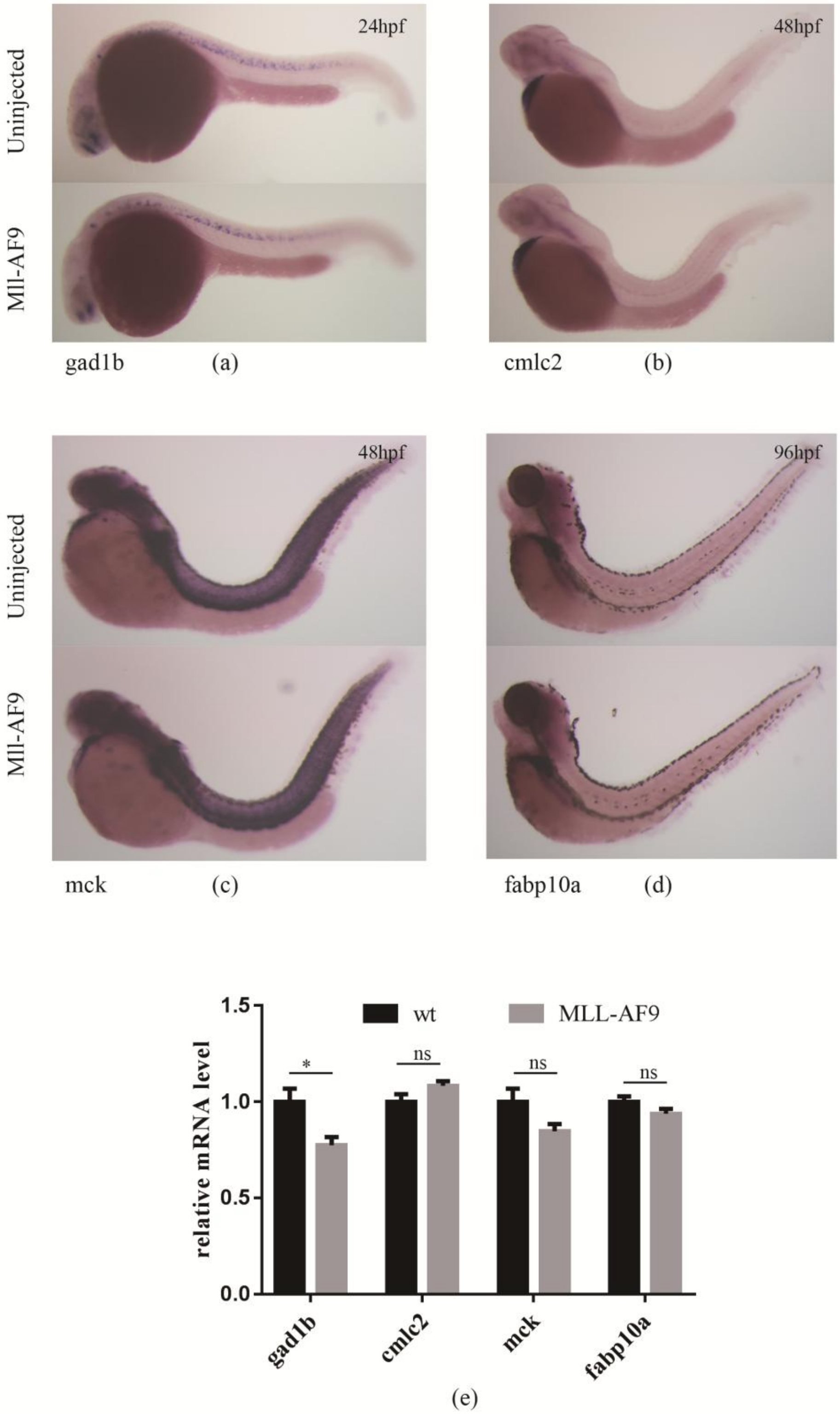

Supplementary Table 1. Summary of primers used in this study

gene	5' Forward 3'	5' Reverse 3'
**MLL-AF9 cloning primers**		
Primers used in pMSCV-neo-MLL-AF9 plasmid	ggactccttctctaggcgccggaa	tagacaaacgcacaccggcctt
Primers used in T3-MLL-AF9-polyA plasmid	ctagcgattacgccaagctc	aaaatttaacgcgaattttaacaaa
**q-RT PCR primers**		
hoxa2b	ggcccaagcagaaccctaat	ccatcagaaatctcgggcga
hoxb5b	tcaggagacccggtacagac	taagtctgctgttgccggtt
pu1	tcggtgtgttaccctcacaa	accatgctctcctcctgttg
csf1r	actcctcaagaatgctgccc	gcttcgttctgacccgtaca
mfap4	ggaaggagaggtttcgctca	tggccagataaagagtcgcc
mpo	gcgccactcgtcagaaaatc	agggttgagcacatgaaggg
lyz	gcctactgggaaagcaggtt	cccgtcatcacaccacttga
csf3r	ggccaggttgtcccaagaaa	gcagtcctcctgaacacaca
fli	caacggatccagagagtcg	ccatgtagccagtatagttcatctg
l-plastin	cctacgtggccaacctctt	catccgcaagatccacatag
hbbe1	tgctctctccaggatgttga	tcacagtcttgccgtgtttc
gad1b	caagaaggcaggagctgttc	gtggcattcacaaacagtgg
cmlc2	ttgagcaatcacaaatacagga	ttatgggcccttttccttct
mck	tcgctgaccacttcctcttt	cttcatgttgccacccttct
fabp10a	ccagtgacagaaatccagca	gttctgcagaccagctttcc
**probe PCR primers**		
lyz	cgtggatgtcctcgtgtgaa	ctttgtttgcgctgctcaca
Hoxa9a	aaccgcaggatgaagatgaaaaag	accgaaggggatagcacagttg

Hoxb5a	aacgcttcgtacagggactc	tatcctccttcttcgggtga
Meis1	catgggctcatccgtcaat	tctcctgtgctaggtgatgc
gad1b	gctgaaatacggggtcagaa	gcaccatccacatgtaacca
cmlc2	gaggcttttggctgcataga	gctgctgatgtgaatgttgaa
mck	ctgtgttgctggtgatgagg	gaacccatggttgtgcttct
fabp10a	gcaggtttacgctcaggaga	tcctgatcatggtggttcct
