# Supplementary material for: Corrigendum to “Human MLL-AF9 Overexpression Induces Aberrant Hematopoietic Expansion in Zebrafish”
Source: Biomed Res Int. 2022 Jan 22;2022:9839650. doi: 10.1155/2022/9839650 (PMC8800627; doi:10.1155/2022/9839650)
Supplement: Supplementary Materials — Original Western blot for MLL in Figure 1(a) in the article and an independent replicate of the same experiment. Original Western blot for GADPH from Figure 1(a). Images of the MLL1 (D2M7U) Rabbit mAb (Amino-terminal Antigen) antibody. Raw mRNA expression levels for Figures 1(b), 2(i)–2(k), 3(e), and 4(e). [file 9839650.f1.zip › Thermo Fisher PageRulerTM 26616, former specification in the laboratory.pdf]

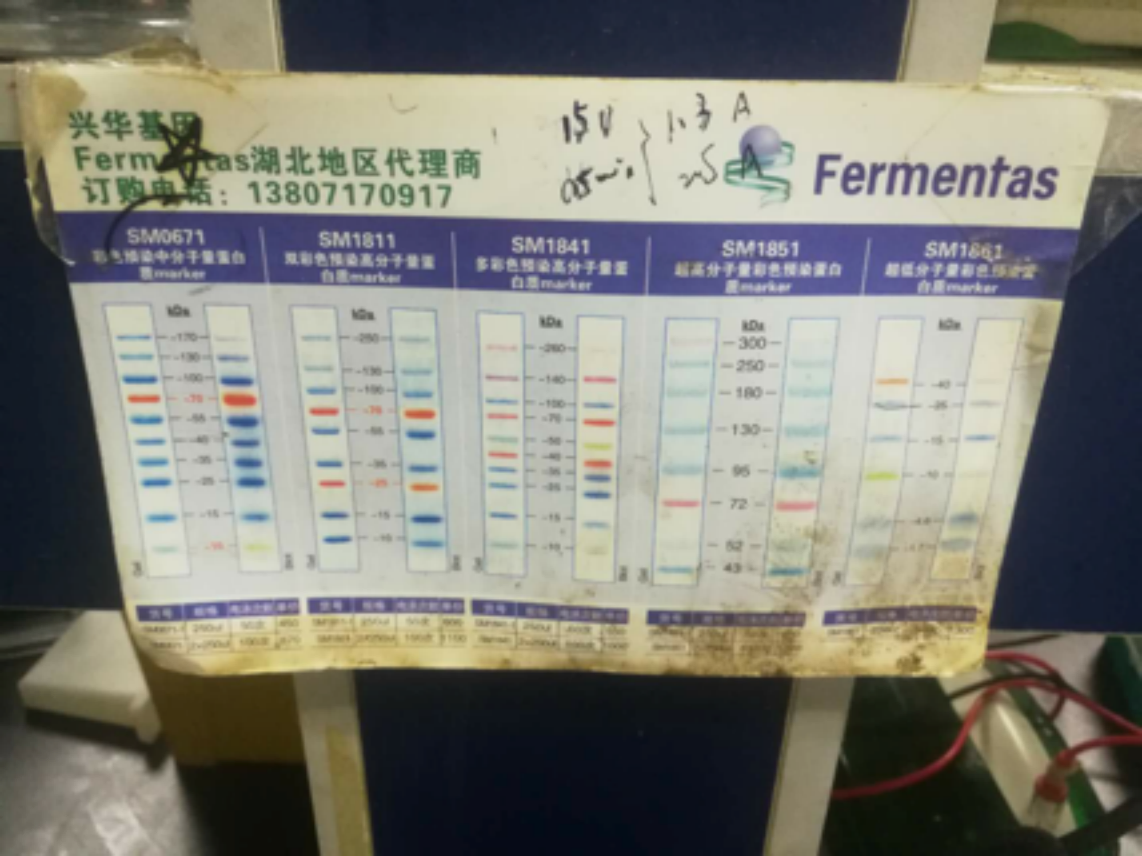

Fermentas湖北地区代理商
订购电话：13807170917
Fermentas
SM0671
SM1811
SM1841
SM1851
SM1861
kDa
300
250
180
130
95
72
52
43
