# Supplementary figures and images for: Corrigendum to “Human MLL-AF9 Overexpression Induces Aberrant Hematopoietic Expansion in Zebrafish”
Source: Biomed Res Int. 2022 Jan 22;2022:9839650. doi: 10.1155/2022/9839650 (PMC8800627; doi:10.1155/2022/9839650)

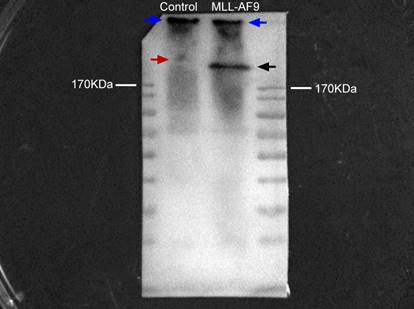

Supplement: Supplementary Materials — Original Western blot for MLL in Figure 1(a) in the article and an independent replicate of the same experiment. Original Western blot for GADPH from Figure 1(a). Images of the MLL1 (D2M7U) Rabbit mAb (Amino-terminal Antigen) antibody. Raw mRNA expression levels for Figures 1(b), 2(i)–2(k), 3(e), and 4(e). [file 9839650.f1.zip › Annotated replicate Western Blot for MLL.jpg]

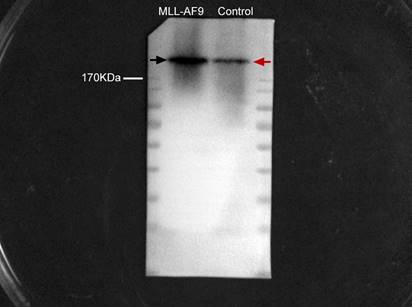

Supplement: Supplementary Materials — Original Western blot for MLL in Figure 1(a) in the article and an independent replicate of the same experiment. Original Western blot for GADPH from Figure 1(a). Images of the MLL1 (D2M7U) Rabbit mAb (Amino-terminal Antigen) antibody. Raw mRNA expression levels for Figures 1(b), 2(i)–2(k), 3(e), and 4(e). [file 9839650.f1.zip › Annotated Western Blot for MLL from Figure 1(a).jpg]

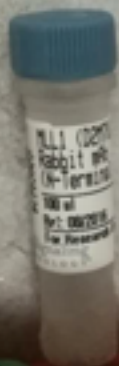

Outlier

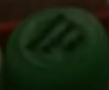

Reverse  
Primer

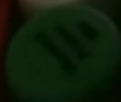

RRI

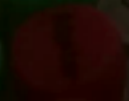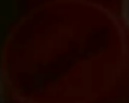

Reverse  
Primer

Reverse  
Primer

Supplement: Supplementary Materials — Original Western blot for MLL in Figure 1(a) in the article and an independent replicate of the same experiment. Original Western blot for GADPH from Figure 1(a). Images of the MLL1 (D2M7U) Rabbit mAb (Amino-terminal Antigen) antibody. Raw mRNA expression levels for Figures 1(b), 2(i)–2(k), 3(e), and 4(e). [file 9839650.f1.zip › MLL1 (D2M7U) Rabbit mAb (Amino-terminal Antigen) antibody in the laboratory 2.pdf]

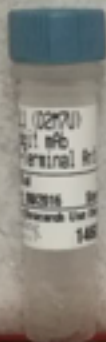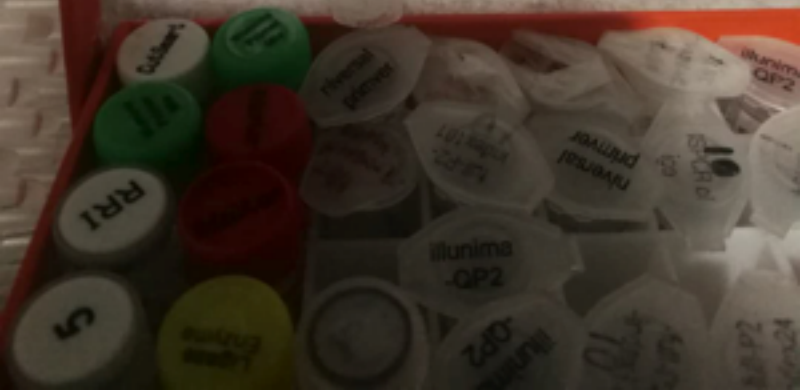

Supplement: Supplementary Materials — Original Western blot for MLL in Figure 1(a) in the article and an independent replicate of the same experiment. Original Western blot for GADPH from Figure 1(a). Images of the MLL1 (D2M7U) Rabbit mAb (Amino-terminal Antigen) antibody. Raw mRNA expression levels for Figures 1(b), 2(i)–2(k), 3(e), and 4(e). [file 9839650.f1.zip › MLL1 (D2M7U) Rabbit mAb (Amino-terminal Antigen) antibody in the laboratory.pdf]

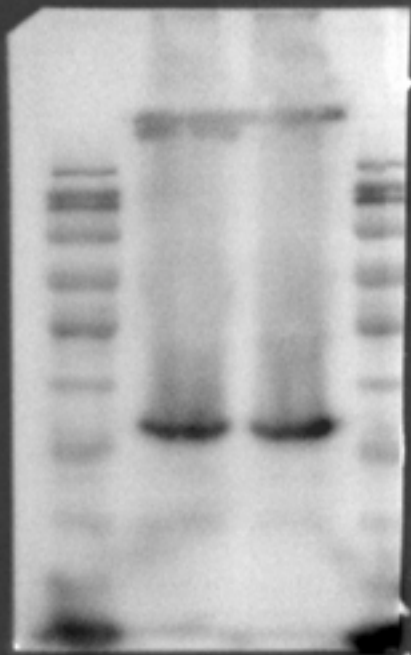

Supplement: Supplementary Materials — Original Western blot for MLL in Figure 1(a) in the article and an independent replicate of the same experiment. Original Western blot for GADPH from Figure 1(a). Images of the MLL1 (D2M7U) Rabbit mAb (Amino-terminal Antigen) antibody. Raw mRNA expression levels for Figures 1(b), 2(i)–2(k), 3(e), and 4(e). [file 9839650.f1.zip › Original Western Blot for GAPDH from Figure 1(a).pdf]

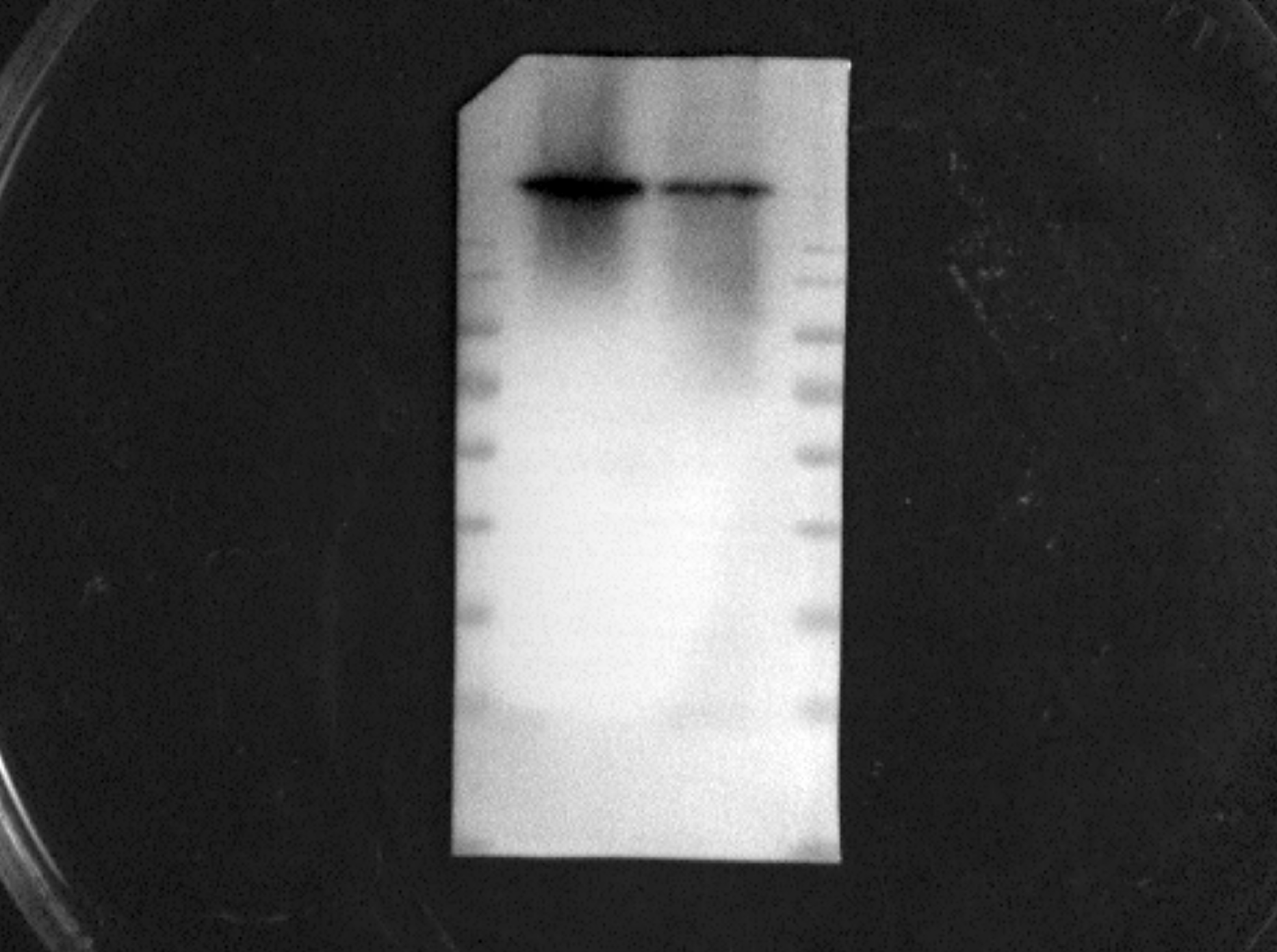

Supplement: Supplementary Materials — Original Western blot for MLL in Figure 1(a) in the article and an independent replicate of the same experiment. Original Western blot for GADPH from Figure 1(a). Images of the MLL1 (D2M7U) Rabbit mAb (Amino-terminal Antigen) antibody. Raw mRNA expression levels for Figures 1(b), 2(i)–2(k), 3(e), and 4(e). [file 9839650.f1.zip › Original Western Blot for MLL from Figure 1(a).tif]

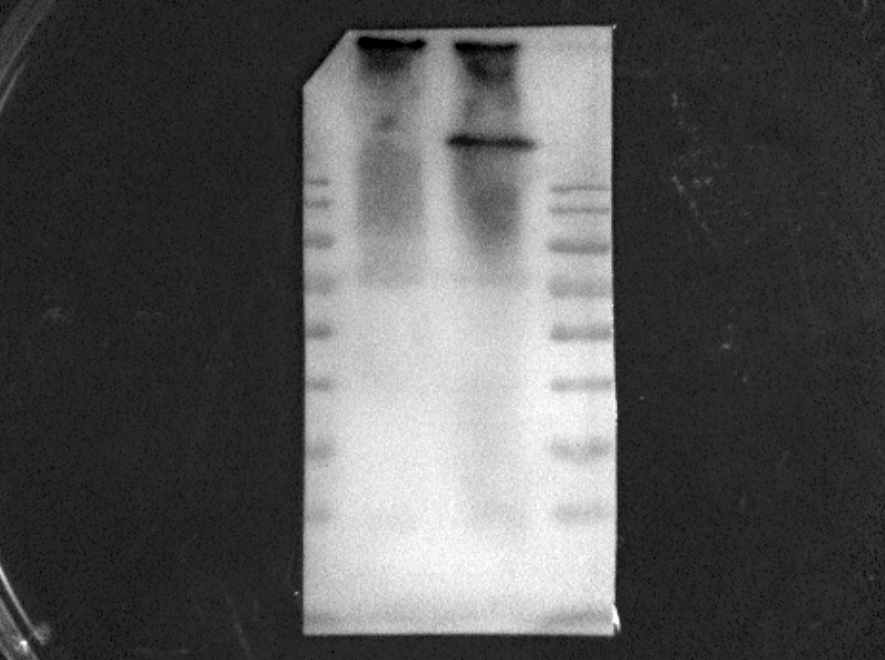

Supplement: Supplementary Materials — Original Western blot for MLL in Figure 1(a) in the article and an independent replicate of the same experiment. Original Western blot for GADPH from Figure 1(a). Images of the MLL1 (D2M7U) Rabbit mAb (Amino-terminal Antigen) antibody. Raw mRNA expression levels for Figures 1(b), 2(i)–2(k), 3(e), and 4(e). [file 9839650.f1.zip › Replicate Western Blot for MLL.tif]

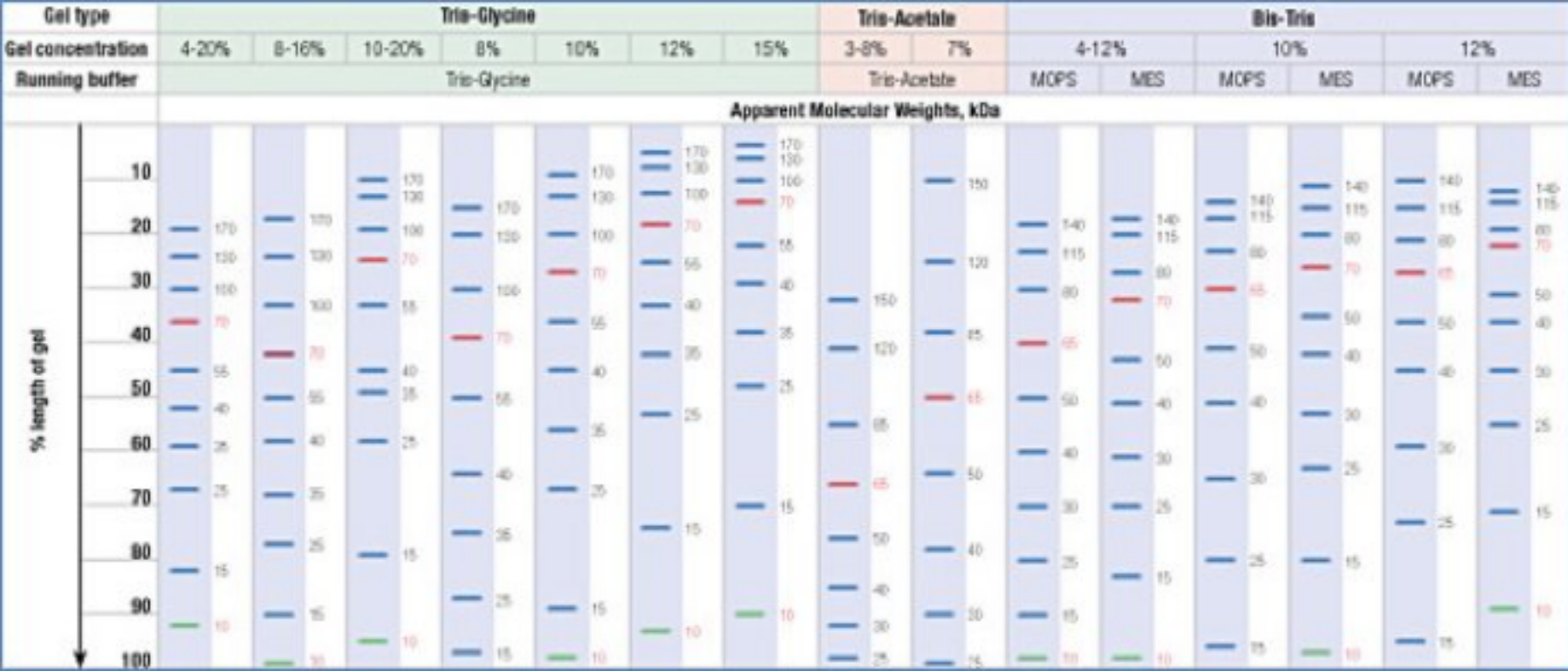

Supplement: Supplementary Materials — Original Western blot for MLL in Figure 1(a) in the article and an independent replicate of the same experiment. Original Western blot for GADPH from Figure 1(a). Images of the MLL1 (D2M7U) Rabbit mAb (Amino-terminal Antigen) antibody. Raw mRNA expression levels for Figures 1(b), 2(i)–2(k), 3(e), and 4(e). [file 9839650.f1.zip › Thermo Fisher PageRulerTM 26616, current specification.pdf]
